# Beyond the Sleep Lab: A Narrative Review of Wearable Sleep Monitoring

**DOI:** 10.3390/bioengineering12111191

**Published:** 2025-10-31

**Authors:** Maria P. Mogavero, Giuseppe Lanza, Oliviero Bruni, Luigi Ferini-Strambi, Alessandro Silvani, Ugo Faraguna, Raffaele Ferri

**Affiliations:** 1 Vita-Salute San Raffaele University, 20132 Milan, Italy; paola_mogavero@libero.it (M.P.M.); ferinistrambi.luigi@hsr.it (L.F.-S.); 2 Division of Neuroscience, Sleep Disorders Center, San Raffaele Scientific Institute, 20127 Milan, Italy; 3 Oasi Research Institute-IRCCS, 94018 Troina, Italy; glanza@oasi.en.it; 4 Department of Surgery and Medical-Surgical Specialties, University of Catania, 95100 Catania, Italy; 5 Department of Human Neurosciences, Sapienza University, 00100 Rome, Italy; oliviero.bruni@uniroma1.it; 6 Department of Biomedical and Neuromotor Science, University of Bologna, Ravenna Campus, 48100 Ravenna, Italy; alessandro.silvani3@unibo.it; 7 Department of Translational Research and of New Surgical and Medical Technologies, University of Pisa, 56100 Pisa, Italy; ugo.faraguna@unipi.it; 8 Department of Developmental Neuroscience, Istituto di Ricovero e Cura a Carattere Scientifico (IRCCS) Foundation Stella Maris, Calambrone, 56128 Pisa, Italy

**Keywords:** wearable sleep monitors, sleep monitoring technology, sleep trackers, polysomnography, actigraphy, sleep stage classification, remote patient monitoring

## Abstract

Sleep is a fundamental biological process essential for health and homeostasis. Traditionally investigated through laboratory-based polysomnography (PSG), sleep research has undergone a paradigm shift with the advent of wearable technologies that enable non-invasive, long-term, and real-world monitoring. This review traces the evolution from early analog and actigraphic methods to current multi-sensor and AI-driven wearable systems. We summarize major technological milestones, including the transition from movement-based to physiological and biochemical sensing, and the growing role of edge computing and deep learning in automated sleep staging. Comparative studies with PSG are discussed, alongside the strengths and limitations of emerging devices such as wristbands, rings, headbands, and camera-based systems. The clinical applications of wearable sleep monitors are examined in relation to remote patient management, personalized medicine, and large-scale population research. Finally, we outline future directions toward integrating multimodal biosensing, transparent algorithms, and standardized validation frameworks. By bridging laboratory precision with ecological validity, wearable technologies promise to redefine the gold standard for sleep monitoring, advancing both individualized care and population-level health assessment.

## 1. Introduction

Sleep is critical for a wide range of physiological and cognitive functions, including memory consolidation [1], metabolic regulation [2], immune function [3], and emotional stability [4]. Disrupted or insufficient sleep has been linked to numerous adverse health outcomes, ranging from cardiovascular disease and diabetes to depression and cognitive decline [2,4,5].

The profound impact of sleep on overall well-being has driven decades of technological innovation aimed at understanding and monitoring sleep quality and architecture. Historically, polysomnography (PSG), considered the gold standard for sleep assessment, has offered comprehensive insights into sleep stages, including wakefulness, rapid eye movement (REM) sleep, and non-REM sleep (N1, N2, N3) [6]. PSG involves the recording of multiple physiological parameters, such as electroencephalography (EEG), electrooculography (EOG), electromyography (EMG), and electrocardiography (ECG), during an overnight stay in a sleep laboratory [6]. While PSG provides rich data, its high cost, complexity, and the artificial environment of the sleep lab limit its practicality for widespread and long-term sleep monitoring. These limitations have necessitated the development of more accessible and convenient alternatives for sleep assessment. Recent advances in wearable technology, including advancements in sensor technology, data processing, and miniaturization, have opened avenues for longitudinal, non-invasive sleep assessment in naturalistic settings, outside the confines of a sleep laboratory [7,8]. These devices, from wrist actigraphy to smart headbands/smartwatches, offer the potential to capture sleep-related data over extended periods, providing a more comprehensive and ecologically valid picture of an individual’s sleep patterns. This review aims to provide an in-depth narrative on the evolution, current state, and prospective future of wearable sleep monitors, exploring the various types of devices, their underlying technologies, and their accuracy in assessing sleep parameters. Furthermore, the review will address the potential clinical and research implications of these emerging technologies, including their role in diagnosing sleep disorders, monitoring treatment efficacy, and advancing our understanding of sleep physiology and its relationship to health and disease.

## 2. Historical Evolution of Sleep Monitoring

### 2.1. Early Foundations: Polysomnography

The rigorous scientific exploration of sleep began in the 1950s, with PSG emerging as the gold-standard method for evaluating sleep disorders and architecture [9,10]. PSG integrates multiple physiological recordings—electroencephalography (EEG) to track brain waves, electrooculography (EOG) for eye movements, electromyography (EMG) for muscle tone, and additional measurements such as electrocardiography (ECG) and respiratory monitoring. This multifaceted approach allow clinicians to distinguish between distinct sleep stages: NREM stages (N1, N2, N3) and REM sleep [6].

Despite its high diagnostic value, PSG has limitations. The method is resource-intensive, requires specialized equipment and expert interpretation, and is conducted in environments (sleep laboratories) that might not reflect an individual’s typical sleep patterns [5]. These factors spurred the search for more user-friendly, cost-effective monitoring methods.

PSG is widely recognized as the gold standard for diagnosing some sleep disorders, according to the International Classification Sleep Disorder 3rd Edition TR (ICSD 3rd Ed-TR 2023) [11], but its effective use demands a high degree of specialized expertise. The assessment and scoring of PSG require professionals who are adept at interpreting a complex array of physiological signals—including EEG, EOG, EMG, ECG, and respiratory patterns—to accurately differentiate sleep stages and identify subtle abnormalities. This intricate process often involves manual scoring that can take several hours per study, contributing substantially to personnel costs. In addition, the sophisticated and high-quality equipment required for acquiring and processing these signals represents a significant financial investment, further restricting PSG to specialized sleep centers. Together, these factors underscore the challenges and resource intensiveness of PSG [12].

### 2.2. The Advent of Actigraphy

Early telemetric mobility recordings in humans paved the way for wrist-worn activity monitors; seminal studies first demonstrated feasibility in free-living conditions [13] and then showed strong correspondence between wrist activity and PSG-derived sleep metrics [14].

In the 1970s, actigraphy emerged as an alternative to PSG. Actigraphy uses a wrist-worn accelerometer to record movement, inferring sleep and wake states from periods of inactivity and activity. Actigraphy allowed for extended monitoring in the patient’s natural environment, making it invaluable for assessing circadian rhythms and sleep patterns over days or weeks [15,16].

Actigraphy is a practical, non-invasive tool that employs wrist-worn accelerometers to record movement patterns over extended periods, enabling the assessment of sleep–wake cycles in natural environments. It is particularly useful for long-term clinical and research applications, including the evaluation of insomnia, circadian rhythm disorders, and large-scale epidemiological studies that would be prohibitively expensive and logistically challenging with PSG [15,17,18].

Several studies have demonstrated that actigraphy correlates well with PSG when it comes to estimating fundamental sleep parameters [19]. For instance, total sleep time (TST), sleep efficiency (SE), and sleep onset latency (SOL) can be measured reliably with actigraphy in individuals with relatively regular sleep patterns. In clinical settings, actigraphy has been validated for monitoring patients with insomnia and circadian rhythm sleep disorders, where its ability to capture longitudinal sleep patterns provides valuable insights into habitual sleep behavior. This capability has been particularly beneficial in studies involving shift workers and individuals with irregular sleep schedules [19,20].

However, while actigraphy excels in quantifying overall sleep duration and general sleep–wake transitions, it has significant limitations [19]. Its reliance on movement data means that it cannot differentiate between various sleep stages—such as REM and non-REM sleep—nor can it reliably detect brief arousals or micro-awakenings that are critical for detailed sleep architecture analysis. For example, in patients who experience minimal movement during periods of wakefulness, actigraphy may erroneously classify these intervals as sleep, leading to an overestimation of sleep quality and duration [20]. Furthermore, the device’s accuracy can be compromised in populations with atypical movement patterns, such as children or individuals with movement disorders.

For instance, in the management of shift-work sleep disorder, actigraphy helps clinicians monitor changes in sleep timing and duration, providing crucial data that informs behavioral interventions and treatment adjustments [21].

Recently, clinical practice guidelines have been established for the use of actigraphy in clinical settings, recommending its application in both the diagnosis and follow-up of certain sleep disorders in both pediatric and adult populations. These include conditions such as insomnia and circadian rhythm disorders [17]. Additionally, recent guidelines extend its use to monitoring adults with sleep apnea syndrome, assessing suspected sleep deprivation in adults, and tracking both adult and pediatric patients prior to undergoing the Multiple Sleep Latency Test (MSLT) [17].

In summary, while actigraphy is a highly valuable tool for assessing general sleep–wake patterns over extended periods, its limitations in resolving the complexity of sleep architecture mean that it cannot fully replace PSG when detailed physiological data are required. Instead, actigraphy is best regarded as a complementary method—ideal for capturing habitual sleep behaviors in large and diverse populations, yet insufficient for diagnosing complex sleep disorders that necessitate in-depth electrophysiological analysis [15,19,20].

It should be noted, however, that modern research-grade actigraphs have advanced well beyond simple accelerometry. Contemporary devices integrate additional sensors, including ambient light and temperature detectors, and employ sophisticated algorithms capable of distinguishing rest-activity patterns with greater precision. These improvements have strengthened actigraphy’s role in the assessment of circadian rhythm sleep–wake disorders, insomnia, and sleep–wake timing in both research and clinical contexts, underscoring its continued value as a complementary and cost-effective monitoring tool.

### 2.3. Transition to Electronic and Digital Monitoring

Advancements in electronic components and digital computing in the late 20th century initiated a revolutionary transition from analog to digital sleep monitoring. In the analog era, sleep data were recorded on paper or magnetic tape, with limited channels and resolution, which often constrained the accuracy and scope of physiological recordings. With the advent of digital systems, it became possible to capture high-fidelity, multi-channel physiological data—including EEG, EOG, EMG, and ECG—allowing for a more comprehensive representation of sleep architecture. This digitization not only improved the accuracy of sleep stage scoring by preserving the integrity of the data over time but also paved the way for efficient data storage and sophisticated processing techniques [22].

The introduction of digital recording technologies revolutionized the workflow in sleep laboratories. Early digital PSG systems could standardize data collection through uniform formats, which facilitated remote data transmission and later integration with computer algorithms [23]. Such systems enabled sleep technicians and clinicians to access and analyze sleep studies from remote locations, thereby enhancing diagnostic efficiency and fostering collaborative clinical environments. Moreover, the capacity to integrate computer-based algorithms for automated scoring reduced manual scoring time and inter-scorer variability—issues that were prevalent in the analog era. This automated analysis has significantly contributed to more consistent and objective sleep assessments, which are critical in both research and clinical applications [24,25].

The digital transition also opened up new avenues for large-scale epidemiological studies and long-term sleep monitoring. Enhanced data storage capabilities allowed researchers to amass and process vast quantities of sleep data, leading to a deeper understanding of sleep patterns and disorders across diverse populations. For example, digital PSG has been instrumental in identifying subtle changes in sleep architecture that correlate with various clinical conditions, thereby informing the development of targeted interventions and personalized treatment strategies. These advancements have not only bolstered diagnostic precision but have also enabled the integration of sleep data into broader health informatics platforms, paving the way for innovative telemedicine applications and remote patient monitoring [26].

In summary, the shift from analog to digital sleep monitoring represents a critical milestone in sleep medicine. The enhanced capabilities in data storage, processing, and automated analysis have transformed how sleep is studied and diagnosed. By enabling more precise, efficient, and remote evaluations, digital technologies have laid the groundwork for ongoing innovations that continue to improve both clinical practice and research in sleep science.

## 3. Emergence of Wearable Sleep Monitors

### 3.1. The Consumer-Grade Revolution

The early 2000s marked a transformative period in consumer electronics, heralding the advent of wearable technology that would soon revolutionize personal health monitoring. Pioneering devices such as the Jawbone UP and Fitbit Flex emerged on the market with an emphasis on general wellness and fitness tracking. Originally designed to monitor physical activity, steps, and caloric expenditure, these devices soon incorporated basic sleep monitoring functions that relied largely on accelerometry. This integration enabled them to estimate sleep duration and SE by analyzing movement patterns during the night, thereby extending their functionality beyond daytime fitness tracking [27,28].

By shifting sleep monitoring from clinics to everyday users, these early wearables democratized access to sleep data. Millions of individuals began tracking their sleep in the comfort of their own homes, gaining insights into their nightly rest that were previously accessible only through expensive laboratory-based polysomnography. This accessibility empowered users to monitor their sleep trends over extended periods and to identify irregular sleep patterns or disturbances in real time. For example, a user might observe that on nights following intense physical activity or increased stress, their SE declines, prompting them to adjust their routines accordingly. Such real-world data collection has not only increased individual awareness of sleep hygiene but has also provided a foundation for large-scale epidemiological studies linking sleep behavior with overall health outcomes [27,28].

As consumer interest grew and technology advanced, subsequent iterations of wearable devices evolved to incorporate additional sensors and more sophisticated algorithms. While early models depended primarily on accelerometry, newer devices began integrating heart rate monitors, gyroscopes, and even skin temperature sensors. These enhancements allowed for more detailed assessments of sleep, including estimations of sleep stage transitions—albeit still not matching the precision of clinical polysomnography [7]. Nevertheless, the improvements significantly narrowed the gap between consumer-grade sleep tracking and clinical diagnostics, making these devices valuable tools for both personal wellness and preliminary health assessments [27].

In summary, the early 2000s set the stage for the wearable revolution by introducing devices like the Jawbone UP and Fitbit Flex, which brought basic sleep monitoring capabilities to a mass audience. The integration of accelerometry-based sleep tracking into these consumer electronics not only provided millions with the means to assess their sleep duration and efficiency in real time but also spurred a broader movement toward self-managed health and wellness. This paradigm shift continues to influence both the consumer market and clinical research, as wearable technology evolves to offer increasingly sophisticated insights into sleep health [27,28].

### 3.2. Integration of Multi-Sensor Technologies

Recent wearable devices have evolved far beyond simple step counting, incorporating a suite of sensors that collectively enhance the accuracy and depth of sleep monitoring. Modern wearables commonly integrate photoplethysmography (PPG) for heart rate and heart rate variability (HRV), skin temperature sensors for circadian rhythm assessment, galvanic skin response (GSR) for arousal detection, and high-precision motion sensors (accelerometers and gyroscopes) for activity and posture analysis. By combining these physiological signals, devices can provide a more accurate and comprehensive estimation of sleep architecture, including transitions between light, deep, and REM sleep [29,30,31,32,33,34].

Devices like the Oura Ring, Whoop Strap, and advanced Fitbit models harness this multi-sensor integration to not only measure basic parameters such as TST and SE but also to approximate sleep staging by recognizing the unique physiological signatures of light, deep, and REM sleep. For instance, a study by de Zambotti et al. [35] demonstrated that the multi-sensor approach employed by the Oura Ring yielded sleep measurements that were in reasonable agreement with PSG—the clinical gold standard for sleep assessment. This represents a significant leap from earlier devices that relied solely on accelerometry-based movement detection [36].

The convergence of these diverse sensor technologies into a single wearable device has revolutionized sleep monitoring. It has enabled continuous, long-term data collection in real-world settings, providing valuable insights into individual sleep patterns over extended periods. This rich dataset not only benefits personal health management—by facilitating early detection of sleep disturbances and monitoring the effectiveness of sleep interventions—but also supports large-scale epidemiological studies aimed at understanding the broader implications of sleep on overall health. In summary, the evolution of wearable sleep technology through multi-sensor integration has dramatically enhanced our ability to assess and understand sleep, marking a transformative advancement in both consumer health and clinical research [7].

Despite its clear potential, multi-sensor fusion also poses technical and algorithmic challenges. Combining signals with different sampling rates, noise levels, and motion sensitivity requires careful synchronization, filtering, and feature-level integration to ensure data reliability. Increasing the number of sensors may at times introduce additional noise or computational complexity. Nevertheless, when properly optimized, the complementary nature of multi-sensor data typically enhances accuracy and robustness, as each signal contributes unique physiological information that strengthens overall sleep assessment.

**Figure 1:**
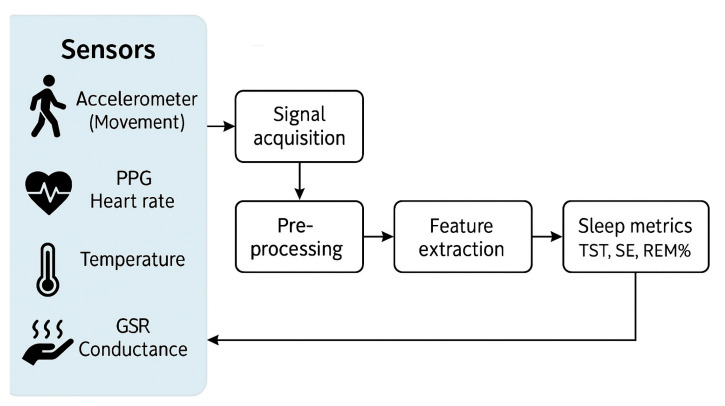
Schematic architecture of a multi-sensor wearable sleep monitor. PPG, photoplethysmography; GSR, galvanic skin response; TST, total sleep time; SE, sleep efficiency; REM, rapid eye movement.

The architecture of a multi-sensor wearable sleep monitor (Figure 1) usually includes sensor signals from accelerometers (movement), PPG (heart rate), temperature, and GSR (conductance) which undergo acquisition, pre-processing, and feature extraction before machine-learning-based classification to derive key sleep metrics such as total sleep time, sleep efficiency, and REM sleep percentage.

### 3.3. Videosomnography for Sleep Monitoring in Infants and Children

Videosomnography has emerged as a valuable tool for sleep monitoring in infants and children, offering a non-invasive and continuous method of assessing sleep patterns. Among the available systems, the Nanit videosomnography system has gained popularity as a leading device for pediatric sleep monitoring, combining high-quality video surveillance with advanced sleep analytics. The Nanit system features a high-definition camera with both day and night vision, enabling uninterrupted sleep monitoring regardless of lighting conditions. It is designed to track and analyze sleep patterns, recording sleep duration, wake times, and overall sleep quality, and generates comprehensive sleep reports to aid parental and clinical decision-making [37,38].

One of the most notable features of the Nanit system is its breathing motion monitoring capability. By detecting subtle chest and body movements associated with respiration, it provides insights into an infant’s sleep safety. Additionally, the device is equipped with built-in environmental sensors that monitor room temperature and humidity, further enhancing its utility in sleep health assessments. The system’s user-friendly mobile app delivers real-time notifications to caregivers’ smartphones, alerting them to significant changes in sleep patterns or environmental conditions. Its ease of setup and intuitive interface make it particularly appealing for home use, bridging the gap between consumer sleep tracking and clinical sleep assessment.

Beyond its practical applications, scientific evaluations have begun assessing the accuracy of videosomnography compared to traditional pediatric sleep monitoring methods. A preliminary study investigated the Nanit Pro’s performance in detecting infant sleep patterns, reporting 97.8% sensitivity in classifying sleep and 60.4% specificity in identifying wakefulness compared to expert-observed scoring [39]. These findings highlight the potential of videosomnography as a supplementary tool for sleep research and clinical monitoring, particularly for populations where traditional wearable sensors may be impractical.

However, the relatively low specificity of videosomnography for wake detection indicates a tendency to overestimate total sleep time and underestimate brief awakenings. In clinical settings, this limitation may affect the assessment of sleep fragmentation in infants, where short arousals and transitional wake episodes are common. For this reason, videosomnographic data should be interpreted alongside behavioral observations, caregiver reports, or complementary physiological measures to ensure accurate evaluation of sleep continuity and quality.

### 3.4. Advances in Algorithm Development and Machine Learning

The true power of wearable sleep monitors lies in their sophisticated data processing algorithms. Advances in machine learning have enabled the development of models that can interpret the complex, multi-dimensional data collected by modern sensors. By training on large datasets—including those derived from PSG studies—these algorithms learn to identify subtle patterns in signals from accelerometers, PPG, and skin temperature sensors, ultimately transforming raw sensor data into clinically meaningful metrics such as TST, SE, and even estimates of specific sleep stages [7].

One of the most significant breakthroughs in this area has been the application of deep learning models. Convolutional Neural Networks (CNNs) are widely used to extract spatial and local features from time-series sensor data. For example, CNNs can process segments of accelerometer and PPG data to detect subtle changes that signify transitions between wakefulness and various sleep stages, such as light, deep, and REM sleep [40]. Complementing CNNs, Recurrent Neural Networks (RNNs), particularly those employing Long Short-Term Memory (LSTM) units, have proven adept at capturing the temporal dynamics inherent in sleep patterns. LSTM networks excel at modeling sequential dependencies—learning how the state in one epoch of sleep influences subsequent epochs—which is critical for accurate sleep staging [41,42].

In addition to deep learning, traditional machine learning algorithms continue to play an important role. Techniques such as Support Vector Machines (SVMs) and Random Forest classifiers have been applied to classify sleep versus wake states based on manually engineered features extracted from sensor signals. These methods have demonstrated robust performance, especially in scenarios where the complexity of deep learning might not be warranted or when computational resources are limited [43]. Moreover, probabilistic models like Hidden Markov Models (HMMs) have been used to capture the stochastic nature of sleep transitions. HMMs estimate the probability of transitions between sleep stages given the observed sensor data, providing a statistical framework that complements both traditional and deep learning approaches—especially in contexts where training data may be limited [42,44].

Despite these advancements, a significant barrier remains: the proprietary nature of many commercial algorithms. Many wearable device manufacturers develop closed-source solutions, which limits transparency and hinders external validation. Without open access to the underlying methods and data, it becomes challenging for researchers and clinicians to independently benchmark performance or fully understand the decision-making processes embedded in these algorithms. This lack of transparency ultimately complicates efforts to standardize and improve wearable sleep monitoring technologies for widespread clinical adoption [7].

Despite these advances, several practical limitations still constrain the full implementation of AI-based analytics in wearable sleep monitors. The restricted battery capacity, limited onboard memory, and processing power of compact devices can hinder continuous real-time analysis. As a result, most commercial systems rely on periodic data transfer to smartphones or cloud platforms, where computationally intensive models are executed remotely. This approach enables the use of complex deep-learning architectures but increases dependence on stable connectivity and raises data privacy considerations. However, recent progress in low-power, edge-computing chips and lightweight neural-network architectures suggests that on-device inference may soon become feasible. Such “edge AI” approaches could enable real-time feedback and adaptive monitoring while minimizing latency, energy consumption, and data transmission requirements.

In summary, the integration of advanced machine learning techniques—ranging from deep learning models like CNNs and LSTMs to traditional methods such as SVMs, Random Forests, and HMMs—has dramatically enhanced the capabilities of wearable sleep monitors. These algorithms enable the extraction of nuanced insights from complex physiological signals, thereby improving the accuracy of sleep staging and overall sleep analysis. However, to realize the full potential of these technologies in both research and clinical settings, greater transparency and standardization in algorithm development are essential.

### 3.5. User-Centric Design and Data Accessibility

Beyond the hardware and algorithms, the success of wearable sleep monitors depends critically on user experience. Advances in user-centric design have led to devices that are not only highly accurate but also comfortable and unobtrusive enough to be worn for extended periods. Modern wearables now feature ergonomically designed bands, lightweight materials, and flexible form factors that minimize discomfort during sleep, ensuring that users are more likely to wear the devices consistently over long durations [45,46].

In addition to physical comfort, the integration of intuitive data visualization platforms into mobile applications has played a pivotal role in enhancing user engagement. These platforms transform complex sleep metrics into easily interpretable visuals and summaries, enabling users to effortlessly track their sleep duration, efficiency, and quality over time. They also offer personalized recommendations and the ability to set sleep hygiene goals, empowering users to make informed decisions about their sleep habits. Such user-friendly interfaces not only promote adherence but also foster a proactive culture of self-monitoring and health management [45].

Moreover, studies have shown that the seamless integration of these features into daily life is key to the widespread adoption of wearable sleep monitors. For example, research by de Zambotti et al. demonstrated that when wearable devices are designed with the end-user in mind—balancing technological sophistication with ease of use—users are more likely to engage regularly with the device, leading to more reliable and actionable sleep data [7]. This emphasis on user experience has been instrumental in transitioning sleep monitoring from a niche clinical practice to a mainstream component of personal health management, underscoring the importance of both hardware design and user-centric software development in the success of modern wearable technologies.

Sustained user engagement remains a key challenge for long-term sleep monitoring. Beyond improving comfort through ergonomic design, several strategies are being explored to promote adherence, including personalized feedback, motivational notifications, gamification features, and the provision of intuitive progress reports that help users visualize their improvements over time. Although missing data may occur when users forget to wear the device, the redundancy inherent in continuous, long-term monitoring greatly attenuates the impact of such gaps. Modern algorithms can interpolate short data losses or flag incomplete nights for exclusion, thereby maintaining the validity and reliability of longitudinal sleep assessments when overall compliance is adequate.

**Figure 2:**
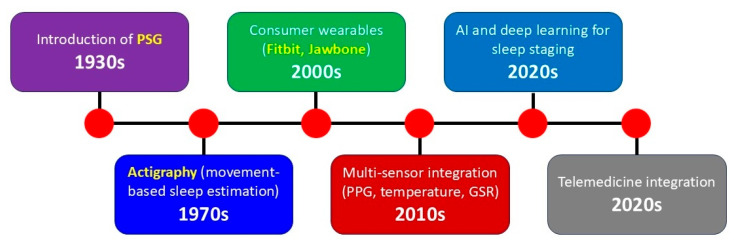
Timeline illustrating key milestones in the evolution of sleep monitoring technologies from the 1930s to the 2020s. PSG, polysomnography; PPG, photoplethysmography; GSR, galvanic skin response.

Figure 2 summarizes the major milestones in the development of sleep monitoring methods and devices. In the 1930s, the introduction of PSG marked the beginning of modern sleep science, allowing simultaneous recording of multiple physiological parameters such as EEG, EOG, and EMG. In the 1970s, actigraphy emerged as a non-invasive, movement-based method for estimating sleep–wake patterns in natural environments. The 2000s saw the rise of consumer wearables (e.g., Fitbit Flex, Jawbone UP), democratizing access to sleep tracking through accelerometry-based devices. In the 2010s, the integration of multi-sensor systems, including photoplethysmography for heart rate monitoring, temperature sensors, and galvanic skin response for electrodermal activity, greatly improved the physiological depth of wearable measurements. The 2020s have been characterized by the application of artificial intelligence (AI) and deep learning algorithms for automated sleep staging, as well as the integration of wearable sleep data into telemedicine and remote patient monitoring platforms.

Table 1 summarizes key wearable devices that have been applied for sleep monitoring, from the first research-grade actigraphs of the 1970s to modern commercial and experimental multi-sensor platforms. The table lists the primary sensing technologies, core physiological or behavioral parameters, and the main strengths and limitations of each approach, illustrating the progressive expansion from movement-based estimation to integrated physiological and biochemical monitoring.

## 4. Validation and Accuracy of Wearable Sleep Monitors

### 4.1. Comparative Studies with Polysomnography

Given that PSG remains the gold standard for sleep assessment, numerous studies have sought to validate wearable devices by comparing their output against PSG-derived sleep metrics. These investigations have primarily focused on evaluating key sleep parameters such as TST, SE, SOL, and wake after sleep onset (WASO). For instance, de Zambotti et al. [47] evaluated the performance of the Jawbone UP against PSG and found good agreement for TST, SE, and WASO. Similarly, a study by Chinoy et al. [48] assessed multiple consumer sleep-tracking devices and reported varying levels of agreement with PSG across these parameters.

Early studies indicated that while wearables could reliably estimate TST and SE, their performance in accurately distinguishing between sleep stages—light sleep, deep sleep, and REM sleep—was less consistent [49]. Wearables frequently overestimated light sleep at the expense of deep sleep and REM, largely due to sensor limitations and proprietary staging algorithms, including differences in accelerometer sensitivity, HRV accuracy, and machine-learning scoring methods [50].

Additionally, many devices struggled to detect brief awakenings or microarousals, which are critical markers for sleep fragmentation and overall sleep quality. For instance, a comparative study involving several consumer-grade devices, including wrist-worn trackers and ring-based sensors, revealed that while some devices showed moderate correlation with PSG for TST, their estimations of REM and deep sleep varied widely [48]. Moreover, the study highlighted that while newer-generation wearables incorporating multi-sensor technology (e.g., combining photoplethysmography with accelerometry) demonstrated improved performance, they still fell short of the precision offered by PSG, particularly for detecting transitional sleep stages and arousals.

The challenge of detecting brief arousals and micro-awakenings represents both a technological and conceptual limitation. These events are traditionally defined by transient EEG activation [6], which cannot be directly recorded by most wearable sensors. Nevertheless, indirect physiological surrogates, such as abrupt changes in heart rate, pulse amplitude (PPG), electrodermal activity, or subtle movement bursts, may provide complementary indicators of arousal-related autonomic activation. Although these proxies cannot fully substitute EEG-based detection, their integration through multi-sensor fusion and machine-learning approaches could pave the way toward a wearable-specific framework for quantifying sleep instability, better suited to real-world monitoring.

These findings underscore the ongoing challenges in achieving PSG-level accuracy with wearable sleep-tracking devices. While they offer convenience and scalability for long-term sleep monitoring, further refinement of sensor technology and algorithmic processing is necessary to enhance their reliability, especially for clinical and research applications [51,52].

### 4.2. Methodological Considerations in Validation

The validity of sleep-tracking wearables depends on multiple methodological factors, each influencing the reliability and accuracy of the recorded data. These factors must be carefully considered when interpreting results from consumer-grade sleep monitors [53].

- Sensor Fidelity: The accuracy of sleep stage classification hinges on the quality of the raw physiological signals captured by wearable sensors. Higher-resolution accelerometers improve motion-based sleep–wake detection, while PPG sensors with greater sampling rates enhance HRV measurements, which are critical for distinguishing between sleep stages. The inclusion of additional biosensors, such as skin temperature and electrodermal activity sensors, may further refine sleep staging by providing complementary physiological markers [54].
- Algorithm Transparency: Many consumer wearables rely on proprietary machine learning algorithms for sleep classification, often trained on limited datasets. While these models can demonstrate strong performance in controlled conditions, the lack of publicly available validation methodologies makes it difficult to assess their generalizability [55]. The use of black-box algorithms also limits cross-study comparisons, as different manufacturers employ distinct training datasets, feature selection processes, and scoring criteria [51].
- Population Variability: Individual differences significantly affect wearable sleep-tracking performance. Factors such as age, sex, cardiorespiratory fitness, and comorbidities (e.g., insomnia, sleep apnea) can alter sensor readings and algorithm predictions. Older adults, for instance, exhibit lower movement levels during sleep, potentially leading to misclassification of wake periods as sleep. Additionally, variations in HRV between individuals can affect the accuracy of sleep stage detection, particularly in devices that rely on PPG-derived HRV metrics.
- Environmental Influences: External conditions can interfere with sleep-tracking accuracy. Ambient temperature fluctuations may impact skin conductance and pulse wave characteristics, leading to inconsistencies in HRV-derived sleep staging. Background noise and disruptions, such as a partner’s movements, can confound accelerometer-based motion analysis. Even sleep posture plays a role, wrist-worn devices may experience signal attenuation if the user sleeps with their arm tucked under their body, potentially reducing sensor contact quality and affecting data reliability [54].

A comprehensive validation approach should account for these methodological considerations, integrating multi-sensor data fusion, diverse population sampling, and standardized testing conditions to improve the robustness of wearable sleep-tracking technologies.

### 4.3. Recent Improvements and Emerging Standards

Recent research has focused on refining algorithms through larger training datasets and incorporating cross-validation with clinical PSG. Some studies now report improved accuracy in detecting sleep stages, especially when multiple sensor modalities are combined. However, there is still a recognized need for standardized validation protocols that can be adopted industry-wide. Collaborative initiatives among academia, industry, and regulatory agencies are underway to establish benchmarks that ensure wearable devices meet acceptable accuracy thresholds for both clinical and consumer applications [54,55]. It is worth noting, however, that emerging composite indices such as “sleep score” or “recovery index,” which are not directly derived from polysomnography, should still be interpreted with caution. Current evidence indicates that objective and subjective sleep parameters are often discordant, and these derived metrics may not fully reflect physiological sleep quality. While PSG should remain the reference validation standard, future studies would benefit from integrating additional functional outcomes, such as daytime alertness, cognitive performance, or well-being measures, to better capture the real-world significance of sleep health.

## 5. Clinical Applications

### 5.1. Remote Patient Monitoring and Telemedicine

The integration of wearable sleep monitors into telemedicine platforms offers a transformative potential for remote patient monitoring. For individuals with sleep disorders such as insomnia, circadian rhythm disorders, sleep apnea, or restless leg syndrome, continuous monitoring outside the laboratory setting can provide clinicians with a more representative picture of sleep patterns over time. This real-world data enables more informed clinical decisions, personalized treatment plans, and timely interventions. For example, in patients with insomnia, wearables can detect patterns such as prolonged sleep latency, frequent nighttime awakenings, or reduced total sleep time over several consecutive nights. These data may trigger a clinical alert or review, prompting evidence-based interventions such as cognitive behavioral therapy for insomnia, reinforcement of sleep hygiene measures, or adjustments in behavioral and environmental strategies. Similarly, recent interventions using smartphone-based virtual agents have demonstrated that improving sleep regularity, as metric wearable devices are particularly suited to measure objectively over long time windows and with significantly more rigor than traditional sleep diaries, can lead to significant reductions in sleep complaints and associated mental health conditions [56]. Moreover, the ability to monitor sleep remotely is especially beneficial for patients in rural or underserved areas where access to sleep laboratories is limited [57]. It is also valuable for individuals who face mobility challenges, such as those with dementia, making traditional in-lab sleep studies difficult [58].

### 5.2. Personalized Medicine and Predictive Analytics

Wearable sleep monitors contribute significantly to the shift toward personalized medicine. By integrating sleep data with other physiological parameters such as heart rate variability, physical activity, and even stress indicators, clinicians can tailor interventions to an individual’s unique profile. Predictive analytics driven by machine learning may one day forecast episodes of sleep disturbance or even predict related cardiovascular events. Such proactive measures could improve outcomes in patients with chronic conditions where sleep quality is a critical factor [59].

While sleep stage analysis remains central to wearable-based sleep monitoring, it alone may not capture the full physiological complexity underlying sleep disorders or comorbid conditions. Integrating additional biosensors, such as those capable of measuring heart rate, respiration rate, peripheral oxygen saturation, skin temperature, and even biochemical markers like glucose, lactate, or ketones, could substantially improve the diagnostic and monitoring potential of wearable systems. Recent studies have demonstrated prototype devices combining electrochemical and optical sensors to simultaneously record metabolic and autonomic indices alongside sleep architecture. For example, in 2023 Charlton et al. [60] highlighted how photoplethysmography-based wearables can extend beyond cardiac and respiratory monitoring to broader physiological applications, while Vural et al. [61] reviewed emerging fluid-based biosensors capable of detecting metabolites such as glucose and lactate in sweat, enabling continuous metabolic assessment. These multimodal sensing platforms enable a more comprehensive evaluation of sleep physiology, linking sleep disruption to systemic metabolic or cardiovascular alterations. In the future, such integrated biosensing and analytics could allow clinicians to move beyond descriptive sleep staging toward physiologically informed, precision diagnostics for sleep and circadian health.

### 5.3. Large-Scale Epidemiological Research

The widespread adoption of wearable devices offers a unique opportunity for large-scale epidemiological studies. Aggregated data from millions of users are already revealing how modern lifestyles, work schedules, and socio-economic factors influence sleep quality, with implications for public health policies and interventions [62,63]. Major initiatives such as the UK Biobank [64] and the U.S. All of Us Research Program [65] have incorporated wearable technology to collect detailed activity and sleep data from large cohorts, exemplifying how these tools can advance precision medicine and population health research. Nevertheless, it is important to acknowledge that participants using consumer-grade wearable devices tend to be overrepresented in higher socioeconomic strata and specific age groups, potentially introducing selection bias into large-scale datasets. To improve representativeness, future research should prioritize targeted recruitment of underrepresented populations, apply statistical weighting to adjust for demographic imbalances, and integrate wearable-derived information with data from traditional cohort and clinical studies. Such strategies will help ensure that findings and subsequent health recommendations derived from wearable-based research are generalizable to the broader population.

### 5.4. Integration with Other Health Monitoring Systems

Wearable sleep monitors are increasingly being integrated into broader health monitoring ecosystems. When combined with data from smartwatches, fitness trackers, and even electronic health records, these devices contribute to a more holistic view of an individual’s health. For example, integrating sleep metrics with dietary and exercise data can help create personalized wellness programs that address multiple facets of health simultaneously [66].

## 6. Conclusions

**Figure 3:**
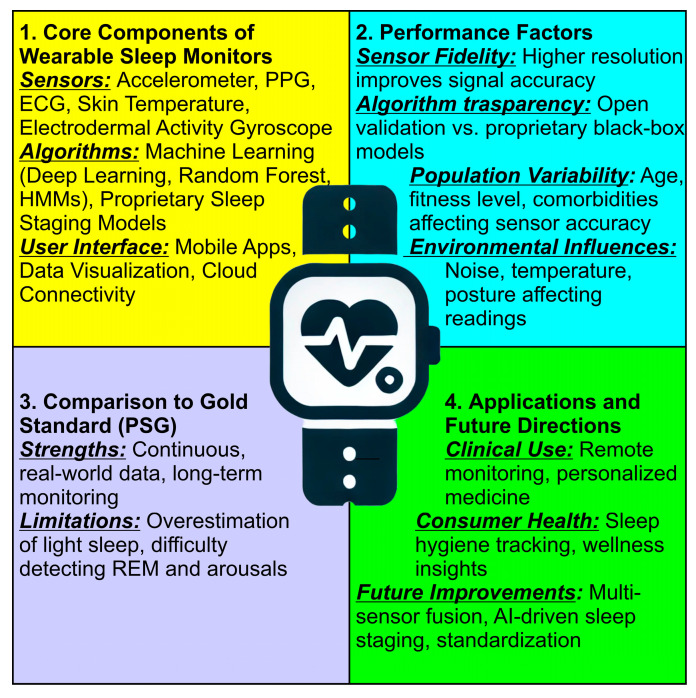
Structured synthesis of key factors influencing the validation and accuracy of wearable sleep monitors. At the center of the figure, a smartwatch icon symbolizes wearable sleep tracking technology, representing the integration of these key factors in real-world applications.

Figure 3 provides a structured synthesis of key factors influencing the validation and accuracy of wearable sleep monitors. The sections are color-coded to highlight different aspects:

1. Core Components of Wearable Sleep Monitors (Yellow Section) lists the fundamental elements of wearable sleep tracking devices, including sensors, algorithms, and user interfaces; covers the role of accelerometers, PPG, ECG, skin temperature, and electrodermal activity in monitoring sleep, and mentions machine learning models and proprietary sleep staging approaches;
2. Validation Factors (Blue Section) highlights the essential criteria for assessing wearable sleep monitor accuracy; includes sensor fidelity, algorithm transparency, population variability, and environmental influences, and emphasizes how external factors like temperature, noise, and posture affect data reliability;
3. Comparison to Gold Standard PSG (Purple Section) discusses how wearables compare to polysomnography (PSG), the clinical gold standard; lists the strengths of wearables, such as continuous real-world monitoring, and identifies limitations, including the overestimation of light sleep and difficulty in detecting REM sleep and arousals;
4. Applications and Future Directions (Green Section) explores potential applications in clinical settings and consumer health; mentions advancements such as multi-sensor fusion, AI-driven sleep staging, and standardization, and highlights the role of wearables in remote monitoring and personalized medicine.

The evolution of sleep monitoring illustrates the rapid technological advances that have transformed our approach to understanding sleep. Wearable sleep monitors provide a unique opportunity to collect continuous, real-world data in a non-invasive manner, thereby complementing traditional clinical methods. While significant challenges remain in terms of sensor accuracy, algorithm transparency, user compliance, and regulatory standardization [67], the potential benefits for both individual health management and large-scale public health research are immense.

The integration of advanced sensors, machine learning, and big data analytics will likely drive further innovations, making wearable sleep monitoring an indispensable tool in the future of personalized medicine. Interdisciplinary collaboration among scientists, clinicians, and regulatory bodies is critical to overcoming current limitations and unlocking the full potential of these technologies. As our understanding of sleep deepens, wearable devices will not only help optimize individual sleep hygiene but also contribute to broader strategies for improving overall health and well-being across diverse populations.

In this context, a “fully solved problem” in wearable sleep monitoring would not necessarily entail perfect concordance with PSG-derived sleep staging. Rather, it would be defined by clinical and functional equivalence, when wearable-derived metrics can reliably predict health outcomes, guide therapeutic decisions, and demonstrate measurable improvements in patient well-being. The ultimate goal is therefore not to replicate laboratory data, but to achieve validated, outcome-driven utility in real-world sleep health management.

Ultimately, the path toward establishing a new gold standard in sleep monitoring lies in bridging the gap between clinical polysomnography and real-world wearable technologies through transparent algorithms, standardized validation frameworks, and longitudinal, ecologically valid data collection. As wearable technologies continue to evolve, their convergence with advanced analytics and open validation pipelines positions them to redefine the gold standard of sleep monitoring itself, transforming it from a laboratory-based assessment into a continuous, personalized, and accessible tool for precision sleep medicine.

## Figures and Tables

**Table 1:** Representative wearable devices for sleep monitoring: technologies, measured parameters, and main features. Adapted from Kripke et al., 1978 [14], de Zambotti et al., 2019 [7], and Yoon & Choi, 2023 [8].

Device/Platform (Representative Model)	Approx. Era	Primary Sensing Technology	Measured Parameters	Main Advantages	Main Limitations
Early wrist actigraphs	1970s	Piezoelectric accelerometer	Total sleep time (TST), sleep efficiency (SE), sleep onset latency (SOL)	Non-invasive, inexpensive, allows long-term home monitoring	Cannot differentiate sleep stages; misclassifies quiet wake as sleep
Mini-Motionlogger (Ambulatory Monitoring, Inc. AMI; Ardsley, NY, USA), Actiwatch (Philips Respironics, Inc.; Murrysville, PA, USA)	1990s−2000s	Triaxial accelerometer ± ambient-light sensor	Activity counts, sleep–wake cycles, circadian phase	Widely validated; incorporates light exposure data	Limited staging resolution; motion artifacts in low-movement conditions
Fitbit Flex (Fitbit; San Francisco, CA, USA), Jawbone UP (Jawbone, AliphCom, Inc.; San Francisco, CA, USA)	Early 2010s	3-axis accelerometer	Sleep duration, SE, bedtime/wake time	Broad consumer accessibility; continuous use	Proprietary algorithms; low staging accuracy
Oura Ring (Oura Health Oy; Oulu, Finland)	2015–present	Photoplethysmography (PPG), temperature, 3-axis accelerometer	TST, SE, HR/HRV, light/deep/REM staging	Multi-sensor integration; good HRV precision	Underestimates wake; algorithm not transparent
Whoop Strap (WHOOP, Inc.; Boston, MA, USA), Garmin Vivosmart (Garmin International, Inc.; Olathe, KS, USA)	2018–present	PPG, galvanic-skin-response (GSR), temperature, accelerometer	Sleep stages, HRV, recovery index	High-frequency sampling; cloud analytics	Sleep staging less accurate than PSG; proprietary scoring
Dreem Headband (Dreem/Rythm SAS; Paris, France), Muse S Headband (InteraXon Inc.; Toronto, ON, Canada)	2018–present	Dry-EEG, PPG, accelerometer	EEG-based sleep stages, HR, respiration	Direct EEG signal; near-PSG accuracy	Less comfortable for long-term home use
Apple Watch Series 6+ (Apple Inc.; Cupertino, CA, USA)	2020–present	PPG, accelerometer, SpO_2_	Sleep duration, HR/HRV, oxygen saturation	Large user base; seamless integration with health platforms	Simplified staging; restricted raw-data access
Nanit System (video-based) (Nanit, Udisense Inc.; New York, NY, USA)	2020s	Computer-vision motion & respiration analysis	Sleep–wake patterns, respiratory motion	Non-contact, infant-friendly	Low wake specificity; dependent on lighting/environment
Experimental biochemical patches	2020s	Sweat-based biochemical sensors (electrolytes, cortisol) + temperature	Stress biomarkers, circadian trends	Adds metabolic insight; expanding research field	Not yet validated for clinical sleep staging

PSG = polysomnography; PPG = photoplethysmography; HR = heart rate; HRV = heart-rate variability; GSR = galvanic skin response; SpO_2_ = peripheral oxygen saturation.

## Data Availability

No new data were created or analyzed in this study.
